# Eating Event Recognition Using Accelerometer, Gyroscope, Piezoelectric, and Lung Volume Sensors

**DOI:** 10.3390/s24020571

**Published:** 2024-01-16

**Authors:** Sigert J. Mevissen, Randy Klaassen, Bert-Jan F. van Beijnum, Juliet A. M. Haarman

**Affiliations:** 1 Department of Human Media Interaction, University of Twente, 7522 NB Enschede, The Netherlands; r.klaassen@utwente.nl (R.K.); j.a.m.haarman@utwente.nl (J.A.M.H.); 2 Department of Biomedical Signals and Systems, University of Twente, 7500 AE Enschede, The Netherlands; b.j.f.vanbeijnum@utwente.nl

**Keywords:** automatic dietary monitoring, eating event detection, piezoelectric sensor, accelerometer, gyroscope, respiratory inductance plethysmography, multi-class classification

## Abstract

In overcoming the worldwide problem of overweight and obesity, automatic dietary monitoring (ADM) is introduced as support in dieting practises. ADM aims to automatically, continuously, and objectively measure dimensions of food intake in a free-living environment. This could simplify the food registration process, thereby overcoming frequent memory, underestimation, and overestimation problems. In this study, an eating event detection sensor system was developed comprising a smartwatch worn on the wrist containing an accelerometer and gyroscope for eating gesture detection, a piezoelectric sensor worn on the jaw for chewing detection, and a respiratory inductance plethysmographic sensor consisting of two belts worn around the chest and abdomen for food swallowing detection. These sensors were combined to determine to what extent a combination of sensors focusing on different steps of the dietary cycle can improve eating event classification results. Six subjects participated in an experiment in a controlled setting consisting of both eating and non-eating events. Features were computed for each sensing measure to train a support vector machine model. This resulted in $F_{1}$ -scores of 0.82 for eating gestures, 0.94 for chewing food, and 0.58 for swallowing food.

## 1. Introduction

### 1.1. Background

Overweight and obesity, defined as having a BMI of over 25 or 30 respectively, is a worldwide problem with an increasing number of cases each year. The percentage of people with overweight or obesity rose from about 29.3% to 39% from 1980 until 2016 [1,2]. Obesity has been associated with cardiovascular disease, type-2 diabetes, osteoarthritis, asthma, and several types of cancer [3,4,5,6]. The number of years of life lost due to being overweight is 3.3 years, and can reach up to 7.6 years of life lost for men and up to 10.3 years of life lost for women in case of severe obesity [7]. Overweight and obesity are one of the biggest avoidable causes of death [8].

Dieting, losing weight, and maintaining long-term weight loss is challenging [9]. Consuming fewer calories than are combusted is considered the best tactic [10,11]. Gaining insight into the current eating behaviour of a person is a first step in accomplishing better health. Professionals continue to use conventional methods such as logbooks or apps for this purpose. These ask the user to manually report their food intake throughout the day. While recall-based food diaries offer a decent estimate of calorie intake for groups, they fall short in accurately estimating an individual’s daily food intake. The method is susceptible to both under- and over-estimation [10,12,13], and is a time-consuming process [14]. Because eating is a routine activity, it is not uncommon for food diary entries to be forgotten [15]. A person might drink a glass of cola after sports, eat a piece of candy on the way to the bus, or receive a piece of cake from a colleague. These are situations that can be forgotten or estimated incorrectly. Additionally, over time people may experience diminishing motivation to continue recording food intake [16]. All of these factors can contribute to inaccurate food intake registration.

**Figure 1:**
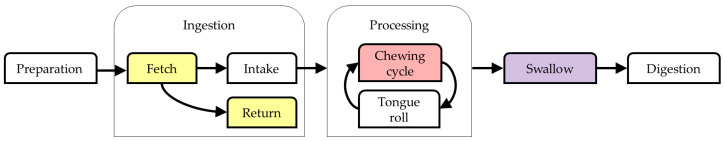
A simplified scheme of the different eating stages from Schiboni and Amft [15]: food preparation, ingestion, processing, swallowing, and digestion. The colours indicate where the different sensors used in this research operate in the eating cycle: yellow for the accelerometer and gyroscope, orange for the piezoelectric sensor, and purple for the respiratory inductance plethysmographic sensor.

The field of automatic dietary monitoring (ADM) aims to streamline and objectify the monitoring of eating habits, focusing on the timing, type, and quantity of food consumed by individuals. In ADM, sensors measure these aspects automatically and continuously, aiming to make the subject a passive element in the food intake registration process. Ideally, the individuals being measured should have no impact on the entries within a food diary in a fully operational ADM system. This ensures that the ADM system does not disrupt eating habits and offers an accurate portrayal of their entire daily food consumption. The initial stage in the ADM process involves the detection of eating events, enabling entries in existing methods to be logged more objectively. Only after implementing event recognition can ADM extend its functionality to determine the type and quantity of food consumed. Sensors designed to detect eating events can function at various stages of the dietary activity cycle. Figure 1 illustrates the dietary activity model, showing distinct phases from food preparation to digestion. The food must first be prepared, for example by cooking or cutting. Subsequently, the food is ingested into the mouth, typically with a hand-to-mouth movement. Next, the food is processed in the mouth by chewing and moving the bolus around. Finally, the food is digested over several days. Measurements can be performed at each of the steps in this process to determine when, what, and how much food is consumed. However, due to the diverse ways in which different types of food are prepared, ingested, processed, and digested, detecting all eating events can be a challenge.

### 1.2. Related Work

**Inertial Measurement Unit**

Fortuna et al. [17] experimented on three participants to detect hand-to-mouth eating gestures, achieving an $F_{1}$ -score of around 0.82. A range of activities were conducted in this experiment, including walking, writing, picking up objects, eating manually or using a fork, and drinking. Fallmann et al. [18] reached an $F_{1}$ -score of 0.79 with only an accelerometer in an experiment with a variety of eating and non-eating related actions using a hidden Markov model. In total, fourteen complex actions were performed. Kyritsis et al. [19] reached an $F_{1}$ -score of 0.78 with a commercially available IMU and a combination of an SVM and a discrete hidden Markov model. Eight participants ate a lunch of their preference, often consisting of a starter, a salad, and a main course. The IMU was worn on the wrist of the arm that was used to hold the fork. Although no other instructions were provided, the amount and variety of non-eating events were limited.

**Piezoelectric Sensor**

Farooq et al. [20] conducted an experiment to detect chewing motions with a piezoelectric sensor, reaching an $F_{1}$ -score of 0.96. The experiment with ten participants consisted of sitting, using a phone, talking, reading out loud, walking, and eating a slice of pizza and a granola bar while walking. A support vector machine (SVM) was trained for the multi-class classification. Fontana et al. [11] attempted to detect chewing motions with a piezoelectric sensor on the jaw. In an experiment, seven test subjects were instructed to talk, walk, read, and eat a meal according to their food preference. SVM was used to classify these activities, whith an $F_{1}$ -score of 0.90 being reached.

**Respiratory Inductance Plethysmographic Sensor**

Tatulli et al. [21] conducted an experiment with five test subjects wearing a respiratory inductance plethysmographic (RIP) sensor vest consisting of two belts for food swallowing detection. The experiments were carried out in a strictly controlled setting with speaking, swallowing food, and regular breathing, and achieved an $F_{1}$ -score of 0.78. Dong et al. [22] used a single RIP sensor belt in a strictly controlled environment to analyse breathing characteristics in order to detect the swallowing of food in six test subjects. The participants were allowed to bring their lunch based on their dietary preferences. An SVM model was trained, which reached an $F_{1}$ -score of 0.73.

### 1.3. Proposed Sensor System

The utilization of a push-button to mark instances of food consumption, observed in the majority of related studies, may have potentially impacted eating behaviours and consequently affected the gathered data. The majority of experiments took place within controlled laboratory settings. Eventually, ADM needs to be implemented in an environment where a great variety of events take place. Consequently, any proposed sensor system must be sufficiently resilient to accommodate these various activities. Eating behaviours are not confined; consuming an apple differs from eating a sandwich or using a spoon. Despite the potential for these sensors to be implemented in real-life scenarios, their outcomes might be influenced by events resembling eating activities. For instance, the jaw movement involved in chewing food resembles non-eating jaw movement during conversation, and an eating gesture might resemble actions such as a person scratching their chin. However, the absence of an eating gesture before a jaw movement indicates that the movement is related to speaking rather than to chewing food. Integration of data from these sensors across various stages of the dietary activity model depicted in Figure 1 can enhance detection scores. Each of the sensors focuses on a different step in the dietary activity model from Figure 1:

- An accelerometer and gyroscope to detect eating gestures in the ingestion step.
- A piezoelectric sensor to detect chewing food in the food processing step.
- An RIP sensor to detect swallowing food in the deglutition phase.

This combination of sensors offers increased resilience against non-eating activities encountered in free-living settings, and offers a more representative evaluation of sensor performance in real-world scenarios. Hence, in this exploratory study, a fresh dataset was gathered using a sensor system comprising a smartwatch with an accelerometer and gyroscope combination along with a piezoelectric sensor and an RIP sensor to detect eating events. The objective was to determine the extent to which the combined use of sensors enhances the results compared to using a single sensor.

## 2. Materials and Methods

An experiment was performed in a controlled setting. This was followed by data processing and window segmentation in preparation for training a support vector machine model aimed at detecting eating gestures, chewing food, and swallowing food.

### 2.1. Experimental Setup

**Figure 2:**
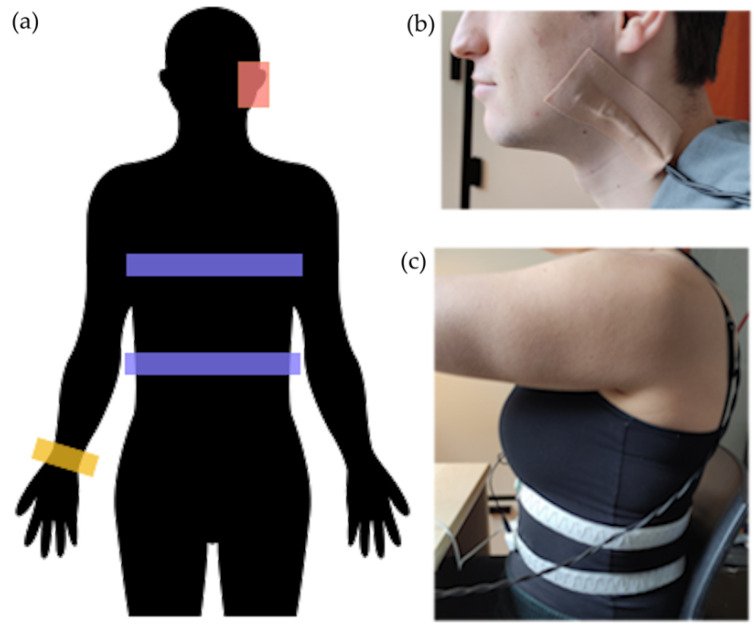
The experimental setup of the sensors. (**a**) Illustration of the sensor locations, with red showing the location of the piezoelectric sensor, purple the two belts of the respiratory inductance plethysmographic sensor, and yellow the smartwatch worn on the wrist. (**b**) The piezoelectric sensor worn by a test subject, attached to the angle of the mandible of the jaw with tape. (**c**) The belts of the RIP sensor worn by a test subject.

A Huawei Watch 2 equipped with both an accelerometer and a gyroscope was worn on the right arm, and was linked to a WebSocket with an average sampling rate of 83 Hz. The piezoelectric sensor (LDT0-028K from TE Connectivity Sensors) was sampled at a frequency of 204 Hz. The sensor was attached to the mandible of the jaw with tape, which was found to be the optimal position [23,24]. The RIP sensor from Ben Bulsink Innoveren met Elektronica consists of two belts worn just above the navel and at the inferior part of the sternum. The belts are designed to measure the circumference of both the abdomen and the ribs. When combined, these measurements provide an estimation of the total lung volume. The RIP sensor was sampled at 6.2 Hz. The sensor setup can be seen in Figure 2.

An experiment was performed on six healthy test subjects. The subjects’ characteristics can be found in Table 1. The experiment took place in a controlled environment, where the subjects were directed to remain stationary except when performing specified actions. As eating activities, the subjects were asked to eat a bowl of yoghurt with pieces of apple using a spoon and to eat pieces of a croissant with their hand. In addition, the participants were instructed to perform a few non-eating actions resembling these eating events, such as scratching the back of their head (eating gesture), random arm movement (eating gesture), and reading aloud from a book (chewing food). To classify swallowing food, non-eating events included regular swallows unrelated to eating and irregular breathing patterns caused by other unrelated actions. The participants were instructed to use their right arm for all eating gesture-related actions. Video recordings were used to create the ground truth. The actions and duration per action can be seen in Table 2. On average, approximately 17 min of data was collected for each test subject.

### 2.2. Data Processing

**Figure 3:**
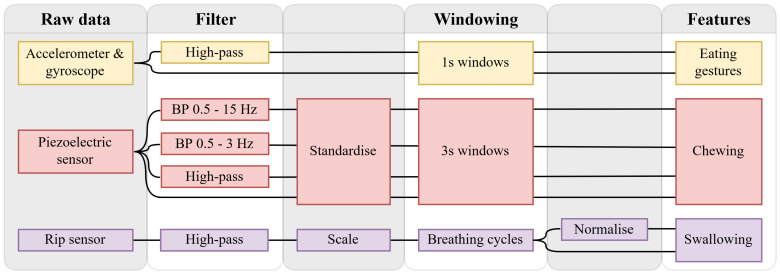
Flowchart explaining the data flow process for the data of the smartwatch, piezoelectric sensor, and respiratory inductance plethysmographic (RIP) sensor. This includes the stages of filtering, scaling, window segmentation, normalization, and feature computation.

Figure 3 illustrates the steps from raw data to feature computation of the three sensors, consisting of filtering the data, standardising or scaling the data, segmenting the data into windows of a specific size, and computing features on these windows.

**Figure 4:**
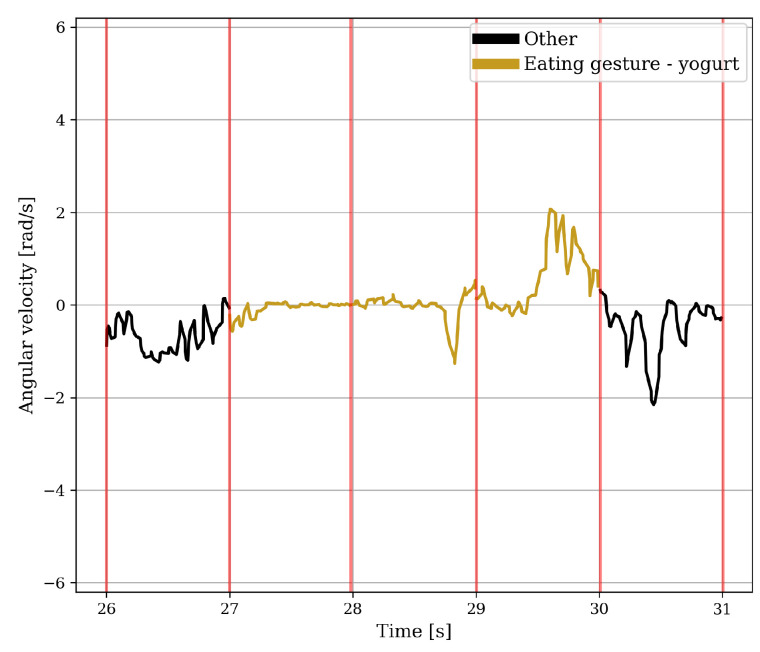
Five windows of the unfiltered angular velocity around the axis of the underarm of a test subject in which the ingestion of a spoonful of yoghurt with apple takes place. The red vertical lines indicate where the data is split into one-second windows.

**Figure 5:**
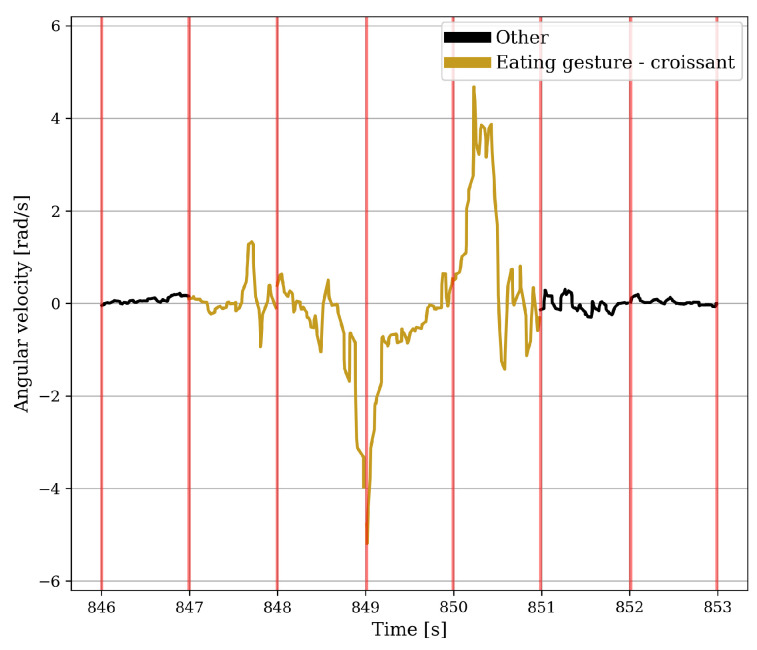
Seven windows of the unfiltered angular velocity around the axis of the underarm of a test subject in which the ingestion of a piece of croissant takes place. The red vertical lines indicate where the data is split into one-second windows.

The accelerometer and gyroscope data were kept in two formats, namely, the raw data and a high-pass filtered data. For the gyroscope, this process was executed to compensate solely for drift, whereas for the accelerometer it was conducted to address both drift and gravitation. The eating gesture features were based on the work by Fallmann et al. [18], Merck et al. [25], and Fortuna et al. [17], in which the data were split into windows of one second. These features were combined with standard statistical features such as the maximum value, minimum value, and standard deviation, resulting in ten gyroscope features and seventeen accelerometer features. Figure 4 and Figure 5 show two instances of rotating around the axis of the underarm for an intake gesture. The angular velocity for the intake gesture of eating yoghurt is constant while the spoon is brought to the mouth. This must be done in a controlled manner to avoid spilling the yoghurt. Because there is no chance of spilling, the angular velocity of the intake gesture for bringing a piece of croissant to the mouth in Figure 5 is more abrupt.

The piezoelectric sensor data were converted into four formats: the original, a demeaned version using a high-pass filter, and two band-pass filtered versions. All signals were standardised to compensate for sensor positioning differences between subjects. The features were based on work by Farooq et al. [20], Fontana et al. [11], and Sazonov et al. [24], in which the piezoelectric sensor data were split into windows of three seconds. These features were then combined with several standard statistical features, resulting in 29 unique features.

**Figure 6:**
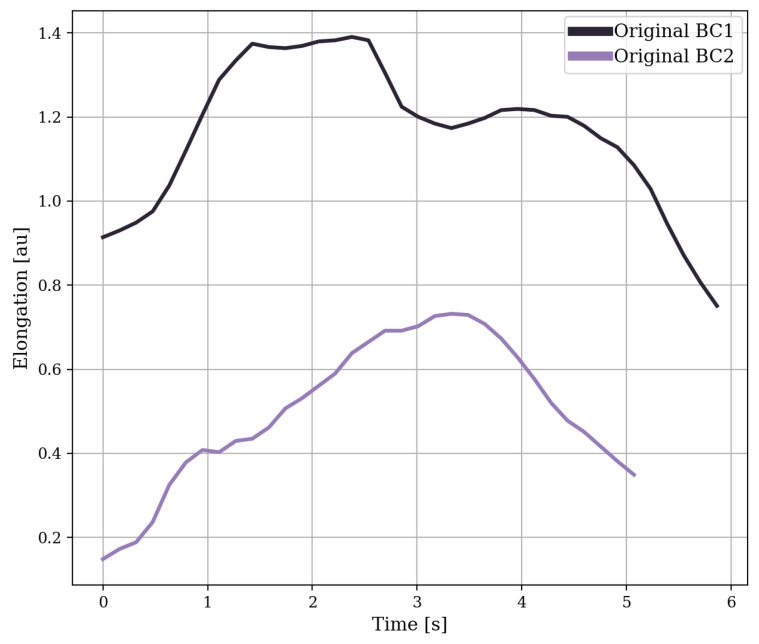
Two examples of breathing cycles (BC) shifted in time to fit a single figure, in which the lung volume is estimated by adding the data from the belts around the abdomen and the ribs.

**Figure 7:**
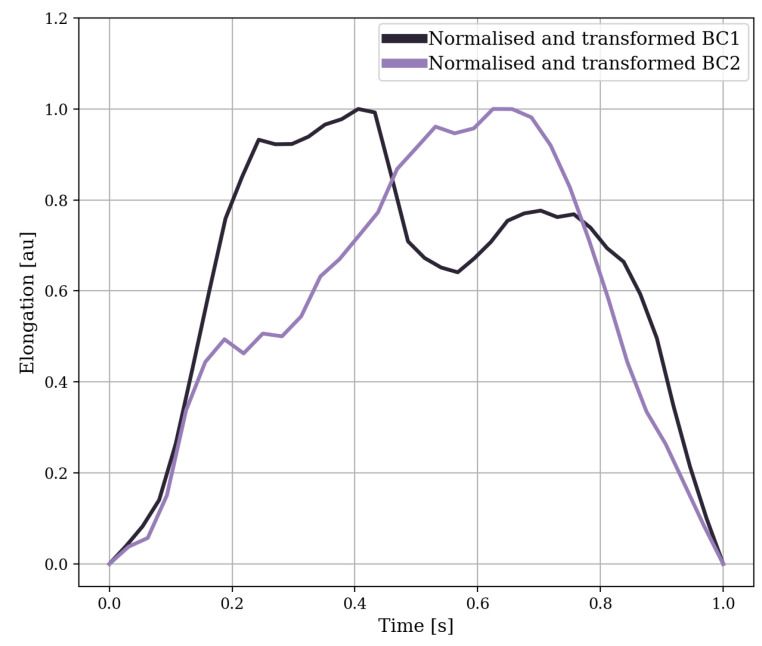
The normalised and transformed version of the breathing cycles from Figure 6. The normalised breathing cycles were normalised in time and amplitude between zero and one and transformed such that $y(t_{0})$ and $y(t_{e})$ were zero.

Because the RIP sensor data showed drift, the data were de-meaned using a high-pass filter. As the RIP sensor did not offer absolute lung volume data, the data were scaled to lung volume of the minimum and maximum inhalation to compensate. The features for detecting swallows, apart from a few statistical features, were primarily based on research by Dong et al. [22,26], in which the RIP data were split into breathing cycles at minimum inhalation. The breathing cycles were normalised and transformed such that the top had a value of one and with breathing cycles starting at t=0 and ending at t=1 , and were further transformed such that $y(t_{0})=0$ and $y(t_{e})=0$ . Figure 6 and Figure 7 show two examples of a breathing cycle before and after normalisation and transformation. The features are based on the normalised (10 features) and non-normalised (26 features) versions of the breathing cycles.

The sampling frequencies of the piezoelectric sensor and the RIP sensor differ by a factor of more than 30. The data were split into windows, then features were computed on these windows, with a set number of features per window; thus, we ended up with a set number of features per time unit unaffected by the sampling rate. A list of all features can be seen in Appendix A.

### 2.3. Feature Vectors

After the features for each window of all signals had been generated, feature vectors were created to function as input for the SVM model. In order to combine the data from the different sensors, the windows and their features must be combined into a single feature vector. For example, if a window from the RIP sensor is to be classified as swallowing and the data from the RIP sensor and the smartwatch are included in the feature vector, the feature vector will consist of features from the windows of the smartwatch and the RIP sensor.

**Figure 8:**
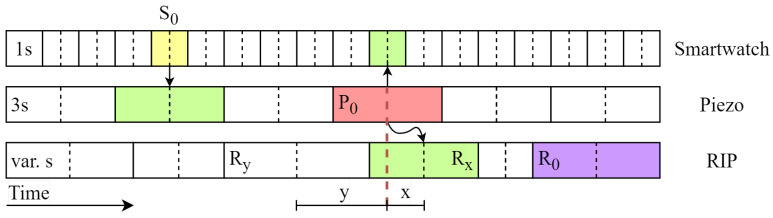
Schematic representation of how the windows of the smartwatch, the piezoelectric sensor, and the respiratory inductance plethysmographic (RIP) sensor are linked, along with their respective features; in this case, all three sensors are included in the feature vector to classify the piezoelectric sensor window $P_{0}$ as chewing. The windows of the other sensors are linked by selecting those windows with the midpoint time closest to $P_{0}$ . Because *x < y*, $R_{x}$ is linked to $P_{0}$ instead of $R_{y}$ . Smartwatch data and piezoelectric sensor data are used in the classification of eating gestures for window $S_{0}$ , linking the green piezoelectric window with a similar midpoint time to the $S_{0}$ window. In the classification of window $R_{0}$ as swallowing, only the RIP sensor is used, incorporating solely the features from that sensor window into the feature vector.

The window sizes of the three sensors are different, as the features of each sensor are based on previous works with those specific window sizes. When incorporating features from multiple sensors into the feature vector, the windows of different sizes must be linked. This is accomplished by finding the midpoint timestamps of each window and finding the closest midpoint timestamp of a window of another sensor. Figure 8 shows a schematic representation of how the windows are linked.

In addition to combining windows of different sensors, features from time-adjacent windows are added to the feature vector for increased time-dependent information. The time-sensitive information encompasses the likelihood of consecutive chewing occurrences and the prerequisite that food must be ingested into the mouth before swallowing. The windows of the three different sensors are of different sizes: the smartwatch sensor features are based on one-second windows, the piezoelectric sensor features on three-second windows, and the RIP sensor features on breathing cycles with varying sizes.

**Figure 9:**
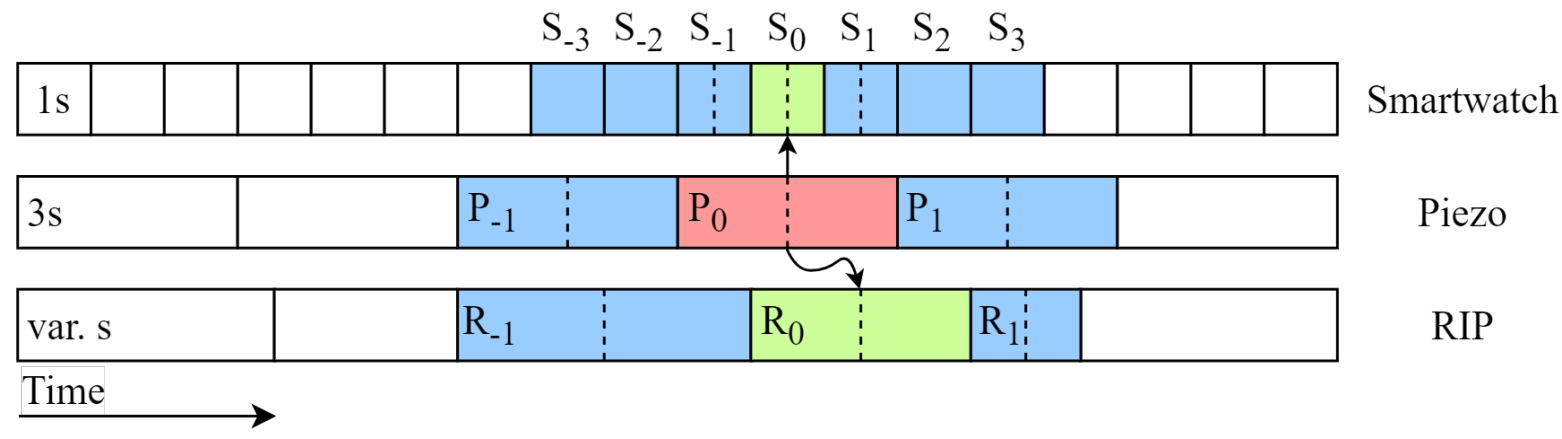
Schematic representation illustrating the extension of feature vectors by appending and prepending windows to the feature vector alongside their respective features. In this specific instance, the piezoelectric sensor window P_0_ is being categorized as chewing and the amalgamation of all three sensors generates a feature vector. First, the green windows R_0_ and S_0_ are linked to P_0_, as described in Figure 8. Then, extra windows are added depending on the feature vector configuration. In this example configuration of 3 s–3 s, three one-second smartwatch windows are added both before ( $S_{-3}$ , $S_{-2}$ , $S_{-1}$ ) and after (S_1_, S_2_, S_3_). Additionally, one three-second piezoelectric sensor window is included before ( $P_{-1}$ ) and after (P_1_) and a single variable-length window of the respiratory inductance plethysmographic (RIP) sensor is added before ( $R_{-1}$ ) and after (R_1_).

Figure 9 illustrates the windows, along with their corresponding features, that are included in the feature vector for the classification of the red piezoelectric sensor window. In this case, data from all three sensors are used to classify the red piezoelectric sensor window. The dashed lines depict the midpoint times of each window. The green windows have the closest midpoint timestamps to the red window, causing the green windows of the other sensors to be linked to the red window. In this 3 s–3 s configuration, three seconds before the linked windows and three seconds after the linked windows are included.

The number of windows to be added to the feature vector is based on the window size. To ensure equal feature vector sizes, which is necessary for the machine learning algorithms, the average breathing cycle duration of 3.6 s was taken to determine how many RIP windows were to be added to the feature vector. In this 3 s–3 s configuration, three windows of one second each are added on both sides of the linked smartwatch window, one three-second window is added on both sides of the piezoelectric sensor window, and one window of varying length is added on both sides of the linked RIP window. This results in a feature vector with features of thirteen windows.

Altogether, eleven configurations of feature vector sizes were constructed: 0 s–0 s, 0 s–3 s, 3 s–0 s, 3 s–3 s, 0 s–7 s, 7 s–0 s, 7 s–7 s, 0 s–10 s, 10 s–0 s, 10 s–10 s, and 13 s–13 s.

### 2.4. Classification

Supervised machine learning algorithms were used to classify the eating events, with the annotations derived from the video recording used as the ground truth. A window was linked to an annotation if any overlap occurred. Prior to feature scaling, the windows were divided into a training/testing set ratio of 2:1. Principal component analysis was then applied for dimensionality reduction of the features while retaining 95% of the variance. Similar to previous works [11,20,22], SVM was used for the eating event classification. A randomised grid search was used to find the optimal hyperparameters. The dataset was imbalanced, with only a small proportion of windows linked to eating gestures (8%), chewing (37%), and swallowing (16%) windows. The micro-averaged $F_{1}$ -score was chosen as a performance metric to overcome this problem. The training data was three-fold cross-validated to prevent overfitting.

## 3. Results

### 3.1. Sensor Combinations

**Figure 10:**
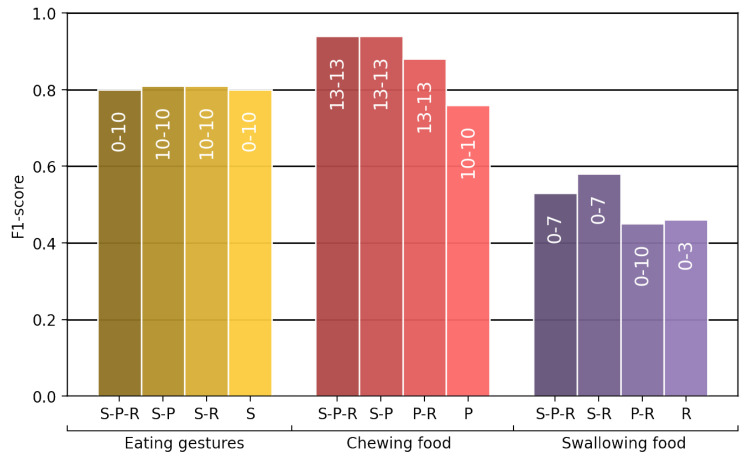
The highest $F_{1}$ -scores of the different sensor combinations for the classification of eating gestures, chewing food, and swallowing food. The characters *S* (smartwatch consisting of accelerometer and gyroscope), *P* (piezoelectric sensor), and *R* (respiratory inductance plethysmographic sensor) denote which sensors were used in each case. The white text indicates which feature vector size was used to acquire the highest $F_{1}$ -score among all feature vectors for that specific sensor combination.

Figure 10 depicts the $F_{1}$ -scores for each of the sensor combinations. The figure displays the peak scores achieved and the corresponding feature vector sizes that contributed to achieving these scores. For the classification of eating gestures, combining sensor data did not improve the results compared to solely using the smartwatch. In the classification of chewing food, the combination of three sensors and the smartwatch–piezoelectric sensor combination achieved the highest $F_{1}$ -scores. The $F_{1}$ -score was 0.94 when combining all three sensors, whereas the $F_{1}$ -score was 0.76 when only the piezoelectric sensor was used. The classifier for the detection of swallowing food achieved less favourable results than the other sensing measures regardless of the sensor combination. Specifically, the combination of all three sensors and the pairing of the smartwatch with the RIP sensor reached higher scores than the remaining two sensor combinations.

### 3.2. Feature Vector Size

**Figure 11:**
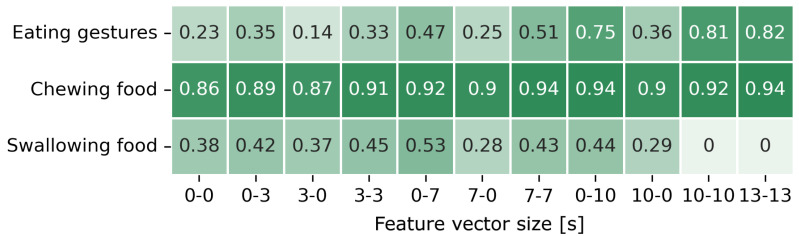
$F_{1}$ -scores for the classification of eating gestures, chewing food, and swallowing food for different feature vector sizes when data from three sensors were combined. The numbers on the x-axis indicate the number of seconds of data that was prepended and appended to the feature vector.

Figure 11 illustrates the $F_{1}$ -scores for the different feature vector sizes of the three sensing measures. For the classification of eating gestures, the highest $F_{1}$ -score of 0.82 was achieved with a feature vector size of 13 s–13 s. In the classification of chewing food, an $F_{1}$ -score of 0.94 was acquired using a 7 s–7 s feature vector size. The classification of swallowing food resulted in the highest $F_{1}$ -score of 0.53 with a feature vector size of 0 s–7 s. The eating gesture and the chewing food classifier showed lower performance when feature vectors solely contained data from the past compared to vectors incorporating both near-past and future data or solely future data. However, for chewing food this difference was relatively small, and this relationship was not found in the classification of swallowing food.

As stated before, each sensor concentrates on a distinct phase of the dietary cycle. Consequently, the data from that particular sensor were consistently incorporated into the sensor combination used for classifying the corresponding sensing measure. This means that the smartwatch sensor was always being used for the classification of eating gestures, resulting in the creation of four sensor combinations per sensing measure.

## 4. Discussion

In determining the extent to which eating events can be recognised, the highest $F_{1}$ -score reached for the detection of eating gestures was 0.82, while for chewing food it was 0.94 and for swallowing food it was 0.58.

The $F_{1}$ -scores achieved for eating gestures in this study resemble the scores reported in previous studies by Fortuna et al. [17], Fallmann et al. [18], and Kyritsis et al. [19], in which similar experiments were conducted.

The classification performance of chewing food using only the piezoelectric sensor was substantially lower than in research by Farooq et al. [20] and Fontana et al. [11]. Those experiments were comparable, with body movement as an extra non-eating event; however, when data from all three sensors were combined, the resulting scores were similar.

In this research, the $F_{1}$ -scores for detection of swallowing food were lower than in the research of Dong et al. [22] and Tatulli et al. [21], irrespective of the combination of sensors used. That research was performed in a controlled environment, similar to this study. The difference in classification scores can be ascribed to several causes, including the lower sampling frequency, the participant instructions, and the different number of participants (three versus six). For example, in Dong et al. [22] the participant pushed a button when swallowing; this can affect the breathing pattern, and could consequently affect the classification of swallowing. The standard frequency for regular breathing cycles is approximately 0.3 cycles per second. Because the RIP sensor data showed drift, the data were de-meaned using a high-pass filter. Although this specific filter was configured slightly above zero Hz, it is plausible that relevant breathing pattern information was lost by applying the filter. This could have affected how the breathing cycles were segmented. Further testing, possibly with another RIP sensor, could help to assess the impact of these parameters on the data.

The results when combining three sensors did not always improve the results compared to using only one sensor. This can be attributed to several factors, such as the type of food consumed, the amount of food consumed, the degree of free-living conditions, the types of sensors, and the means of annotation. The primary objective of this research was to evaluate the extent to which combining diverse sensors could enhance the classification scores, rather than to achieve the highest possible scores. Thus, further testing and development could improve the scores.

In classifying eating gestures, combining three sensors focusing on different steps of the dietary cycle only marginally improved the results compared to solely using the smartwatch sensor. When classifying chewing food, combining data from the three sensors performed consistently better than using only two sensors. Using only the piezoelectric sensor always performed worse, meaning that adding sensors for classifying food chewing improves the results. This was not the case in the swallowing food classification, in which there was much deviation between the different classification scores. Nevertheless, the overall performance of the swallowing food classifier was poorer, leaving unanswered the question of whether combining sensors to classify swallowing food is better.

The features generated from the various sensors were tailored for their respective sensing measures. Thus, the smartwatch features were specifically computed for the classification of eating gestures. Consequently, the features from the other two sensors were primarily centered around chewing and swallowing. Improving sensor combination outcomes might involve designing features that transcend single sensing measures.

The feature vector size is of great influence on the $F_{1}$ -scores of the eating gesture classifiers, with better results for larger feature vectors. The feature vectors using data from the near future provide better results than the feature vectors using data from the past. This observation might be attributed to the experimental conditions, wherein participants were instructed to place their hands back on the table following each bite. This action could serve as a clear indicator for the classifier to detect, potentially contributing to the performance difference. A feature vector size increase shows only minimal improvements for the detection of chewing food, and demonstrates no favourable impact on the classification of swallowing food.

An ADM sensor must meet comfort, aesthetics, mobility, and complexity requirements in order for people to use it throughout the day. Consequently, the gains from wearing multiple sensors need to be substantial enough to outweigh the additional burden imposed by wearing multiple devices. This is especially important in the case of the piezoelectric sensor due to its unaesthetic appearance. On the other hand, the smartwatch can be worn as a regular watch and the RIP sensor can be discreetly positioned beneath clothing, making them more attractive options for users. Based on this observation, coupled with the marginal increase in performance, it can be deduced that the combination of an accelerometer and gyroscope with a piezoelectric sensor and RIP sensor does not yield significant enough improvements to establish itself as a viable option within this setting.

Testing in free-living conditions will introduce a more diverse range of non-eating events, thereby adding a variety of events that closely resemble eating events. Due to the multimodal nature of this setup, with three sensors focusing on different steps of the dietary cycle, it should be easier to distinguish these non-eating events from eating events.

Because the experiment was carried out with a limited sample size, differences in results could have arisen due to chance. The generalisability of the model increases when the sample size is increased, as the data encompass more different types of eating gestures, chewing patterns, and breathing patterns. This can be tested by training a leave-one-subject-out model, in which a test subject is evaluated in the test set without being included in the training set.

## 5. Conclusions

As obesity and overweight become an increasing problem worldwide, healthy eating and dieting have a growing role to play in maintaining healthy weight. Automatic dietary monitoring can play a crucial role in providing objective parameters for eating patterns. An experiment with six test subjects was performed involving eating and non-eating events. Each participant wore a smartwatch containing an accelerometer and a gyroscope on the wrist, a piezoelectric sensor on the jaw, and a respiratory plethysmographic sensor consisting of two belts around the chest and abdomen. By combining three sensors focused on different steps in the dietary cycle, the effect of multimodal data on eating event classification was tested. In addition to this, feature vectors included data from the past and future to encompass the chronological aspect of eating. Combining sensors improved the classification results for chewing. This advantage was not apparent in the classification of eating gestures and swallowing. For each combination of sensors, it must be assessed whether wearing extra sensors outweighs the increased discomfort. To evaluate the advantages of utilizing a multimodal sensing system, further testing in free-living conditions with a variety of food types and non-eating events is essential.

## Figures and Tables

**Table 1:** Subject characteristics.

Subjects	n = 6
Male (%)	50
Age (y)	23.5 ± 1.9
Weight (kg)	76.7 ± 9.6
Heigth (cm)	179.3 ± 10.5

**Table 2:** The actions performed during the experiment along with their respective durations.

Action	Duration
1. Breathe in and out to minimal and maximal lung volume	20 s.
2. Eat a bowl of yoghurt with the right arm. The left hand can be used to hold the bowl in place.	~5 min.
3. Move the right arm in a random fashion above the table	1 min.
4. Sit still + scratch back of head every 10 s	2 min.
5. Read out loud from a book	3 min.
6. Sit still + scratch back of head every 10 s	2 min.
7. Eat a croissant with the right hand	~3 min.
8. Move the right arm in a random fashion above the table	1 min.

## Data Availability

The data generated from the experiment and the source code that support the findings of this study are available from the corresponding author upon reasonable request.

## Abbreviations

The following abbreviations are used in this manuscript:

ADM	Automatic Dietary Monitoring
RIP	Respiratory Inductance Plethysmography
SVM	Support Vector Machine
NBC	Normalised Breathing Cycles
NNBC	Non-Normalised Breathing Cycles

## Appendix A. Features

This is the list of features generated for each window before applying principal component analysis. These features were computed for all signals seen in Figure 3.

### Appendix A.1. Accelerometer and Gyroscope

Most eating gesture features were based on the work of Fallmann et al. [18], Merck et al. [25], and Fortuna et al. [17]

1. Mean
2. Standard deviation
3. Median
4. Correlation
5. Energy
6. Spectral entropy
7. Number of zero crossings
8. Mean time between zero crossings
9. Standard deviation of time between zero crossings
10. Mean magnitude of the gyroscope vector (gyroscope only)
11. Mean magnitude of the derivative of the acceleration vector (accelerometer only)
12. Covariance (accelerometer only)
13. Coefficients of fourth order polynomial fit to each acceleration component with hamming window weighting (accelerometer only, five features per axis)

### Appendix A.2. Piezoelectric Sensor

The chewing food features were based on the work of Farooq et al. [20], Fontana et al. [11], and Sazonov et al. [24]

1. Number of peaks
2. Mean time difference between peaks
3. Standard deviation of the time difference between peaks
4. Number of zero crossings
5. Mean time difference between zero crossings
6. Minimum time difference between zero crossings
7. Maximum time difference between zero crossings
8. Median time difference between zero crossings
9. Standard deviation of the time difference between zero crossings
10. Shannon entropy of the time difference between zero crossings
11. Ratio between the number of peaks and the number of zero crossings
12. Standard deviation
13. Minimum
14. Maximum
15. Mean
16. Median
17. Root mean square
18. Ratio between maximum and root mean square
19. Ratio between root mean square and mean
20. Peak frequency of the fast Fourier transform
21. Standard deviation of the fast Fourier transform
22. Energy
23. Spectral entropy
24. Shannon entropy
25. Shannon entropy of peaks
26. Shannon entropy of derivative at zero crossings
27. Shannon entropy of values nearest to zero crossings
28. Fractal dimension
29. Sample entropy

### Appendix A.3. RIP Sensor

The breathing cycle features for the detection of swallowing food were based on the work of Dong et al. [22,26], and were generated on the normalised breathing cycles (NBC) and the non-normalised breathing cycles (NNBC).

1. Standard deviation
2. Mean
3. Energy
4. Median
5. Root mean square
6. Ratio of the maximum to root mean square
7. Number of peaks
8. First ten fast Fourier coefficients (ten features, NBC only)
9. Hist-60; the amplitude was divided into ten equal intervals, where each datapoint in the breathing cycles fell within one of the intervals, resulting in x values per interval; x was then summed from the bottom up until 60% of the values were included. The value for this feature was the number of the interval at which this 60% was reached. (NBC only)
10. First value (NNBC only)
11. Last value (NNBC only)
12. Maximum (NNBC only)
13. Minimum of inhalation (NNBC only)
14. Minimum of exhalation (NNBC only)
15. Ratio of maximum to minimum inhalation (NNBC only)
16. Ratio of maximum to minimum exhalation (NNBC only)
17. Ratio of minimum to maximum exhalation (NNBC only)
18. Ratio of minimum exhalation to minimum inhalation (NNBC only)
19. Ratio of minimum to maximum inhalation (NNBC only)
20. Ratio of minimum inhalation to minimum exhalation (NNBC only)
21. Duration (NNBC only)
22. Breathing frequency (=1/duration, NNBC only)
23. Duration of inhalation (NNBC only)
24. Duration of exhalation (NNBC only)
25. Ratio of duration of inhalation to duration of exhalation (NNBC only)
26. Ratio of duration of exhalation to duration of inhalation (NNBC only)
27. Absolute breathing cycle duration (NNBC only)
28. Ratio between the duration and average duration of the two neighbouring breathing cycles (NNBC only)
